# Evaluation of the neoadjuvant chemotherapy response in osteosarcoma using the MRI DWI-based machine learning radiomics nomogram

**DOI:** 10.3389/fonc.2024.1345576

**Published:** 2024-03-21

**Authors:** Lu Zhang, Qiuru Gao, Yincong Dou, Tianming Cheng, Yuwei Xia, Hailiang Li, Song Gao

**Affiliations:** ^1^ Department of Medical Imaging, Henan Provincial People’s Hospital, People’s Hospital of Zhengzhou University, Zhengzhou, Henan, China; ^2^ Department of Radiology, Fuwai Central China Cardiovascular Hospital, Zhengzhou, Henan, China; ^3^ Artificial Intelligence Technology, Huiying Medical Technology Co., Ltd, Beijing, China; ^4^ Department of Radiology, Henan Provincial Cancer Hospital, Zhengzhou, Henan, China; ^5^ Department of Orthopedics, Henan Provincial People’s Hospital, People’s Hospital of Zhengzhou University, Zhengzhou, Henan, China

**Keywords:** osteosarcoma, neoadjuvant chemotherapy, MRI, DWI, radiomics, nomogram

## Abstract

**Objective:**

To evaluate the value of a nomogram combined MRI Diffusion Weighted Imaging (DWI) and clinical features to predict the treatment response of Neoadjuvant Chemotherapy (NAC) in patients with osteosarcoma.

**Methods:**

A retrospective analysis was conducted on 209 osteosarcoma patients admitted into two bone cancer treatment centers (133 males, 76females; mean age 16.31 ± 11.42 years) from January 2016 to January 2022. Patients were classified as pathological good responders (pGRs) if postoperative histopathological examination revealed ≥90% tumor necrosis, and non-pGRs if <90%. Their clinical features were subjected to univariate and multivariate analysis, and features with statistically significance were utilized to construct a clinical signature using machine learning algorithms. Apparent diffusion coefficient (ADC) values pre-NAC (ADC 0) and post two chemotherapy cycles (ADC 1) were recorded. Regions of interest (ROIs) were delineated from pre-treatment DWI images (b=1000 s/mm²) for radiomic features extraction. Variance thresholding, SelectKBest, and LASSO regression were used to select features with strong relevance, and three machine learning models (Logistic Regression, RandomForest and XGBoost) were used to construct radiomics signatures for predicting treatment response. Finally, the clinical and radiomics signatures were integrated to establish a comprehensive nomogram model. Predictive performance was assessed using ROC curve analysis, with model clinical utility appraised through AUC and decision curve analysis (DCA).

**Results:**

Of the 209patients, 51 (24.4%) were pGRs, while 158 (75.6%) were non-pGRs. No significant ADC1 difference was observed between groups (P>0.05), but pGRs had a higher ADC 0 (P<0.01). ROC analysis indicated an AUC of 0.681 (95% CI: 0.482-0.862) for ADC 0 at the threshold of ≥1.37×10^-3^ mm²/s, achieving 74.7% sensitivity and 75.7% specificity. The clinical and radiomics models reached AUCs of 0.669 (95% CI: 0.401-0.826) and 0.768 (95% CI: 0.681-0.922) respectively in the test set. The combined nomogram displayed superior discrimination with an AUC of 0.848 (95% CI: 0.668-0.951) and 75.8% accuracy. The DCA suggested the clinical utility of the nomogram.

**Conclusion:**

The nomogram based on combined radiomics and clinical features outperformed standalone clinical or radiomics model, offering enhanced accuracy in evaluating NAC response in osteosarcoma. It held significant promise for clinical applications.

## 1 Introduction

Osteosarcoma is the most common primary bone malignancy, representing 20% of all primary bone cancers (1). This cancer mainly impacts children and adolescents, especially those aged 12 to 18. It is the third most common cancer in this age group, following leukemia and lymphoma, significantly threatening the health and well-being of young individuals (2). Since the 1970s, with the advent and application of neoadjuvant chemotherapy (NAC), the five-year survival rate for patients has risen from 20% to 70%, markedly extending life expectancy (3). The rate of post-chemotherapy tissue necrosis in osteosarcoma is considered the “gold standard” for prognostic evaluation and determines the feasibility of limb-sparing surgery. However, this “gold standard” can only be obtained post-surgery and is not applicable for pre-surgical decisions on limb-sparing operations.

Currently, preoperative assessment of NAC efficacy primarily relies on clinical evaluations, including changes in tumor size, pain scores, and other symptoms or signs, as well as traditional radiological assessments focusing on morphological changes like tumor boundaries and size. Although these methods are somewhat informative, they lack measurable and standardized criteria and fail to precisely gauge chemotherapy effectiveness. Radiological evaluations, including dynamic contrast-enhanced (DCE)-MRI and 18F-FDG PET/CT, offer valuable morphological and functional insights (4, 5). These studies typically employ mean values to represent the entire tumor, potentially overlooking the heterogeneity of the tumor. Yet, these imaging modalities are currently deficient in highly specific standardized parameters. Diffusion-weighted imaging (DWI) is a straightforward, non-invasive functional imaging technique that has been identified as promising in recent studies. The apparent diffusion coefficient (ADC) value, in particular, has shown significant potential in indicating NAC efficacy in cancers such as osteosarcoma (6–9). However, the considerable heterogeneity of tumors may compromise the reliability of these findings, not meeting the rigorous standards required by precision medicine. Radiomics, combining quantitative image analysis with machine learning, identifies diagnostic features and constructs models that provide more precise clinical diagnosis and treatment information. This study investigates the potential of a nomogram model, based on MRI DWI radiomics and clinical features, to assess the effectiveness of NAC in osteosarcoma patients.

## 2 Materials and methods

### 2.1 Patients and dataset

**Figure 1:**
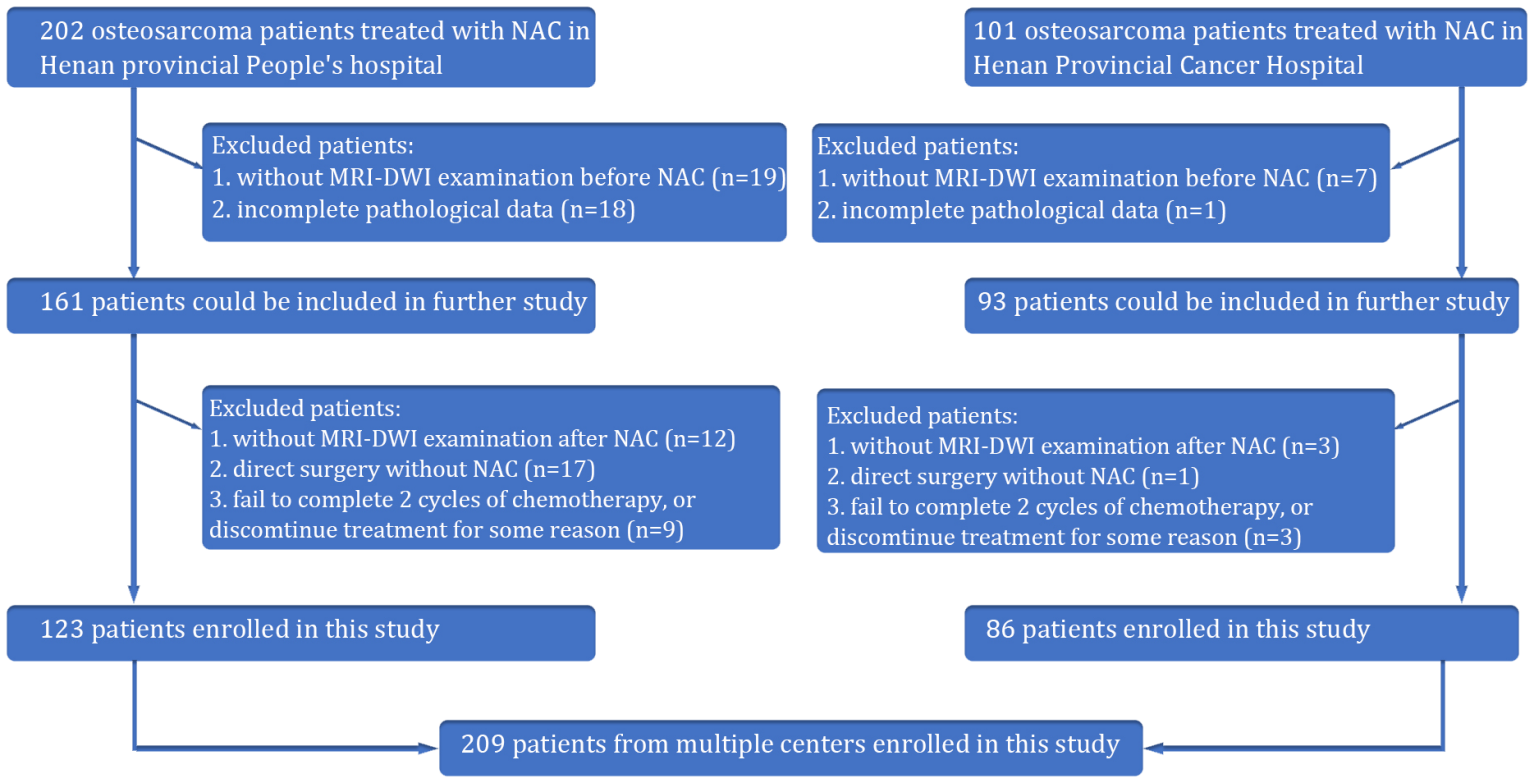
The flow chart showing the inclusion and exclusion criteria of this retrospective study.

Our retrospective study received approval from the Institutional Review Board of Henan provincial People’s hospital, and the requirement for patient informed consent was waived. This was a multicenter retrospective study of osteosarcoma patients from two Chinese hospitals. From January 2016 to January 2022, the information of patients with osteosarcoma in the Henan provincial People’s hospital and Henan Provincial Cancer Hospital was collected based on the following inclusion criteria: φprimary osteosarcoma confirmed by histopathological analysis of tissue samples obtained through biopsy or surgery; κ administration of preoperative neoadjuvant chemotherapy (NAC) with accompanying standard MRI and DWI imaging performed before chemotherapy. Exclusion criteria: φabsence of surgical intervention, which precluded assessment of tumor necrosis rates; κMRI images compromised by severe artifacts that affected diagnostic clarity. Following surgery, the Huvos grading system was utilized (10). Patients exhibiting a necrosis rate of 90% or higher were categorized as pathologically good responders (pGRs), while those with less than 90% were considered non-pGRs. A total of 209 patients were ultimately included in this study (**Figure 1**).

The diagnostic and therapeutic protocols for all participants conformed to the expert consensus on the Clinical Diagnosis and Treatment of Typical Osteosarcoma (11). The administered NAC regimen included doxorubicin (ADM) at 60 mg/m^2^, cisplatin (DDP) at 100 mg/m^2^, methotrexate (MTX) at 10-12 g/m^2^, and ifosfamide (IFO) at 10 g/m^2^. The chemotherapy protocol entailed four rounds, commencing with ADM (D1-D3), followed by DDP (D4), two successive doses of MTX (D1), and concluding with IFO (D1-D5). Patient progress was closely monitored after each treatment cycle, with NAC response typically evaluated after completing a cycle.

### 2.2 Clinical and pathological data

Clinical data were meticulously gathered from medical records, including age, tumor size, tumor anatomical location, presence of pathological fractures, and surgical stage as defined by Enneking’s criteria for osteosarcoma (12).

### 2.3 MRI examination

All MRI images were acquired from two 3.0 T MRI scanners, one model being the GE Discovery 750w (GE Healthcare, USA) and the other the Magnetom Trio (Siemens, Germany), with consistent scanning parameters across both machines. Appropriate surface coils were chosen based on the scanning area, such as an eight-channel knee coil or an eight-channel body soft tissue coil. Routine scans included T1-weighted axial imaging (TR 700 ms, TE 12 ms), proton density-weighted imaging with fat suppression (PDWI-FS) in transverse, sagittal, and coronal planes (TR 4000 ms, TE 83 ms), and diffusion-weighted imaging (DWI) using a single-shot spin-echo planar echo sequence (SE-EPI) with parameters of TR 6000 ms, TE 70 ms, FOV 400 mm × 300 mm, slice thickness of 5 mm, interslice gap of 1 mm. The diffusion sensitivity coefficient b-values were set to 0 and 1000 s/mm^2^, with diffusion-sensitive gradient fields applied along the X, Y, and Z axes.

### 2.4 Imaging findings and radiomics analysis

**Figure 2:**
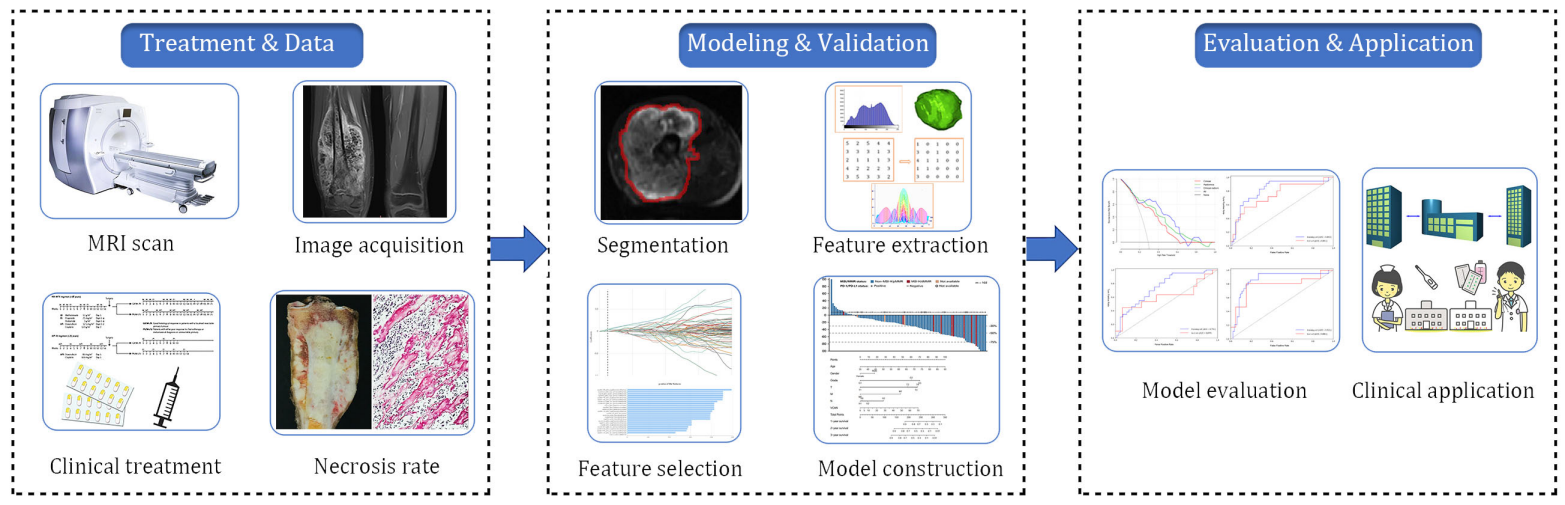
The flow chart of radiomics analysis.

Radiomics analysis was conducted on MRI-DWI sequences, which involved image acquisition and segmentation, feature extraction, feature selection, model construction, and model prediction evaluation (**Figure 2**). Raw image data from the enrolled patients were imported into the Siemens Verio Workstation 3.0 for post-processing. Two radiologists with 9 and 10 years of experience, blinded to clinical and histopathological details, delineated the region of interest (ROI) along tumor margins on DWI images at a b-value of 1000 s/mm². ADC values of the lesions were measured on ADC maps, avoiding areas of intratumoral hemorrhage, calcification, and liquefactive necrosis.

#### 2.4.1 Image normalization and segmentation

To minimize MRI intensity variations, normalization techniques were applied, setting the image intensity relative to the mean and standard deviation. Volumes of interest (VOIs) were precisely traced using ITK-SNAP software (version 3.8.0; http://www.itksnap.org) by an experienced musculoskeletal radiologist, blind to clinical information. These delineations were reviewed by a senior radiologist to resolve any discrepancies in tumor margin identification. VOIs were designed to include the entire lesion, encompassing osseous and soft tissue involvement, and cystic necrosis, while avoiding perilesional edema and vascular structures. In total, 209 VOIs were marked for analysis from the MRI-DWI scans.

#### 2.4.2 Radiomic feature extraction

Using the Radcloud platform (http://radcloud.cn/), 1409 quantitative imaging features were extracted from the MR images. These features were categorized into first-order statistical features (126 descriptors of voxel intensity distributions), spatial geometric features (14 three-dimensional shape and size descriptors), texture features (525 metrics quantifying regional heterogeneity), and transform features (including various transformations). These features were randomly divided into training and testing sets in a 7:3 ratio.

#### 2.4.3 Feature reduction and selection

Features underwent intraclass correlation coefficient (ICC) analysis, with those exceeding an ICC of 0.75 selected for further analysis. Preliminary feature selection was performed using Analysis of Variance (ANOVA) for features with P < 0.05, followed by dimensionality reduction using variance threshold, SelectKBest, and the Least Absolute Shrinkage and Selection Operator (LASSO) method. LASSO was used to balance bias and variance, eliminating redundant or irrelevant features. The final step was to select the most valuable features to construct a radiomics model using logistic regression.

### 2.4.4 Construction of Clinical, Radiomics and Combined Nomogram Models

For the clinical characteristics, we conducted univariate and multivariate analyses sequentially. Features with statistically significant differences in the multivariate analysis were selected for constructing nomograms. Clinical features with significant differences in the univariate analysis were used to develop a machine learning model using logistic regression (LR), RandomForest (RF) and XGBoost. In the process of clinical model building, we used five-fold cross-validation over the training data for model selection and hyperparameter tuning. For Radiomics modelling, we used the same approach as those used in clinical modelling. The combined clinical-radiomics nomogram model was built with the radiomics score and clinical variables used in the clinical model.

### 2.5 Statistical analysis

The Radcloud platform (provided by Huiying Medical Technology Co., Ltd) was used for imaging and clinical data management and radiomics statistical analysis. Additional analyses were conducted using SPSS version 20.0 and MedCalc software version 15.2.2. Independent samples t-tests (two-tailed, p < 0.05) were applied to compare age, tumor dimensions, and ADC values between pGRs and non-pGRs. The Mann-Whitney U test was used in tumor surgical staging, anatomical sites, and pathological fractures. Plot receiver operating characteristic (ROC) curves for traditional imaging (ADC value), clinical model, radiomic model, and clinical-radiomic nomogram model, calculating area under the curve (AUC), sensitivity, and specificity to assess the predictive performance of each model for the effectiveness of neoadjuvant chemotherapy in osteosarcoma. Differences between models are compared using the DeLong test, with p < 0.05 indicating statistically significant differences. Model precision was further examined through radiomics score plots and calibration curves. Decision curve analysis (DCA) was performed to determine the clinical applicability of the predictive models (13).

## 3 Results

### 3.1 Clinical and traditional imaging features

**Figure 3:**
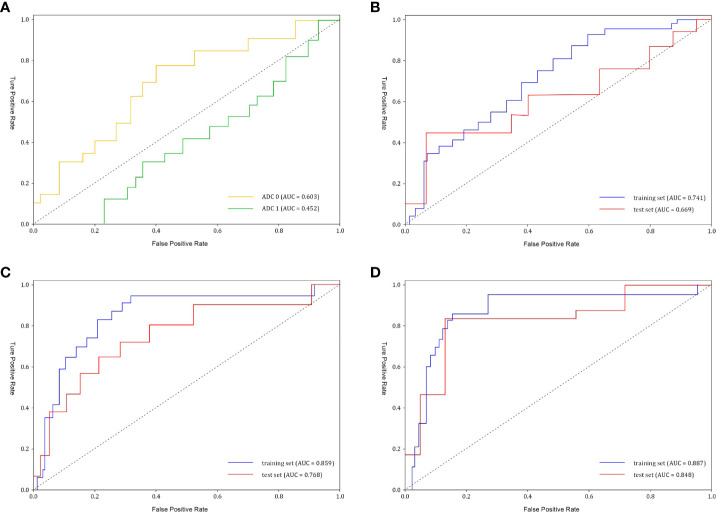
The receiver operating characteristic (ROC) curves of ADC values and different models **(A)** ADC0 represents pre-treatment ADC value (AUC=0.603), and ADC1 represents post-treatment ADC value (AUC=0.452). **(B)** Clinical model performance. The clinical model reached an AUC of 0.669 in the test set. AUC, area under the curve. **(C)** Radiomics model performance. The radiomics model showed an AUC of 0.768 in the test set. **(D)** Clinical-radiomics nomogram model reached an AUC of 0.848 in the test set.

Our study included 209 osteosarcoma patients, consisting of 133 men and 76 women, with a mean age of 16.31 ± 11.42 years, spanning from 6 to 63 years. Among them, 51/209 (24.4%) were identified as pathological good responders (pGRs) to neoadjuvant chemotherapy (NAC), while 158/209 (75.6%) were non-pGRs. Most clinical risk factors, including age, tumor size, pathological fractures, and pathological subtype, did not show a significant statistical difference between the pGRs and non-pGRs (P > 0.05),except for surgical staging (P = 0.0.025) and pathological fracture (P = 0.038).The training set contained a pGR rate of 32/146 (21.9%), and the test set had a pGR rate of 19/63 (30.1%), with no significant difference noted between them (P = 0.484). No significant statistical difference was observed in ADC 1 values between the two (P=0.131). However, the ADC 0 of pGRs was significantly higher than that of non-pGRs (P = 0.007). (**Table 1**) ROC curve analysis indicated that an ADC 0 value ≥ 1.37 × 10^-3^ mm^2^/s achieved a sensitivity of 74.7%, specificity of 75.7%, and AUC of 0.603 (95% CI: 0.458–0.821) in assessing NAC efficacy for osteosarcoma (**Figure 3A**).

**Table 1:** Clinical and traditional imaging features of 209 patients with osteosarcoma.

Features		Training set			Test set	
	pGR	non-pGR	*P* value	pGR	non-pGR	*P* value
Age (mean ± SD)	14.91 ± 8.55	13.42 ± 9.26	0.315	15.99 ± 8.97	14.01 ± 8.95	0.411
Tumor size (mean ± SD) (cm)	13.25 ± 4.89	11.52 ± 5.01	0.411	11.98 ± 4.62	11.69 ± 5.02	0.321
Tumor site			0.523			0.714
thighbone	15	62		10	23	
tibia	14	39		7	17	
humerus	3	13		2	4	
With Pathological fracture	32	114	0.031	19	44	0.038
Surgical stage			0.029			0.025
II	27	85		16	21	
III	5	29		3	23	
ADC 0	1.11 ± 0.03	1.41 ± 0.04	0.024	1.01 ± 0.03	1.37 ± 0.04	0.007
ADC 1	1.43 ± 0.06	1.49 ± 0.03	0.359	1.45 ± 0.07	1.50 ± 0.06	0.131

ADC, apparent diffusion coefficient values; ADC 0 represents pre-treatment ADC values, and ADC 1 represents post-treatment ADC values.

### 3.2 Clinical model

After conducting univariate analyses on all clinical features, we extracted those with p < 0.05, including Surgical Stage and Pathological Fracture, and developed multiple machine learning models. Among these, the Logistic Regression (LR) model achieved the highest AUC (0.669, 95% CI: 0.401-0.826). (**Figure 3B**) We selected Surgical Stage (p < 0.05) from the multivariate analysis to construct the nomogram.

### 3.3 Radiomics model

**Figure 4:**
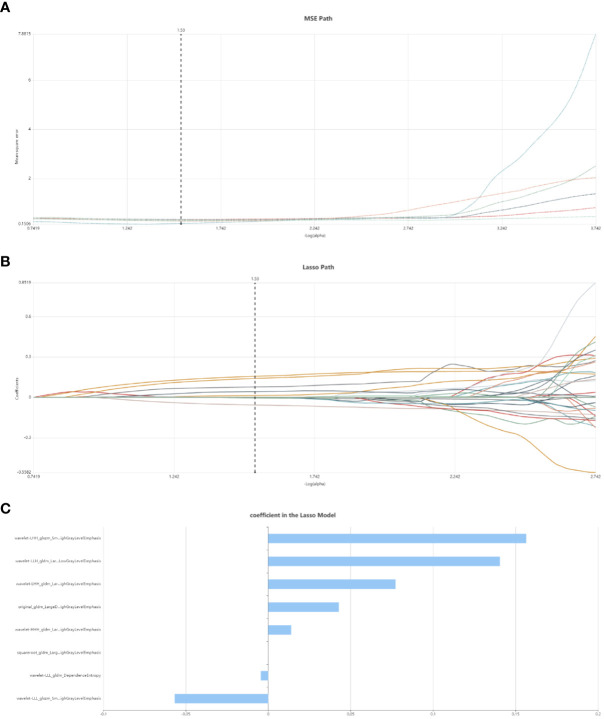
Radiomic feature selection using LASSO regression. **(A)** MSE path, the black solid line is the mean value of Mean Square Error, and the maximum number of iterations is 100. 10-fold cross-test was used to find the optimal -log(alpha) value of 1.53. **(B)** Lasso path, the radiomic features change with alpha value; **(C)** The histogram shows the selected 8 features and their coefficients in Lass model. Using Lasso model, 8 features which are correspond to the optimal alpha value were selected.

Initially, feature stability was assessed using each feature’s intraclass correlation coefficient (ICC), and those with an ICC > 0.85 were retained for further analysis. Out of the total, 445 (61.2%) stable features were selected for further screening. Subsequently, 447 features were chosen using the variance threshold method, which was narrowed down to 82 features by the SelectKBest method. LASSO regression further refined the selection to 82 radiomic features, with 10-fold cross-validation identifying the optimal -log(alpha) value of 1.53 (**Figures 4A, B**). The corresponding coefficients for different features were determined based on the alpha value, ultimately identifying 8 radiomic features strongly correlated with NAC effectiveness (**Figure 4C**). The top 5 features in the LASSO regression being texture features post-wavelet transformation. The selected features, feature groups, and filters are detailed in **Table 2**. Using these 8 features, the radiomics model demonstrated an AUC of 0.859 (95% CI: 0.778–0.909), with a sensitivity of 86.7% and specificity of 71.7% in the training set. In the test set, it achieved an AUC of 0.768 (95% CI: 0.681-0.922), sensitivity of 72.1%, and specificity of 73.4% (**Figure 3C**).

**Table 2:** Description of the selected radiomic features with their associated feature group and filter.

Radiomic feature	Radiomic class	Filter
SmallAreaHighGrayLevelEmphasis	glszm	wavelet-LHH
LargeDependenceHighGrayLevelEmphasis	gldm	wavelet-LHH
LargeDependenceHighGrayLevelEmphasis	gldm	wavelet-HHH
LargeDependenceLowGrayLeve1Emphasis	gldm	wavelet-LLH
Smal1AreaHighGrayLevelEmphasis	glszm	wavelet-LLL
LargeDependenceHighGrayLevelEmphasis	gldm	original
LargeDependenceHighGrayLevelEmphasis	gldm	squareroot
DependenceEntropy	gldm	Wavelet-LLL

GLDM, Gray Level Dependence Matrix; GLSZM, Gray-Level Size Zone Matrix.

### 3.4 Clinical-radiomics nomogram and utility

**Figure 5:**
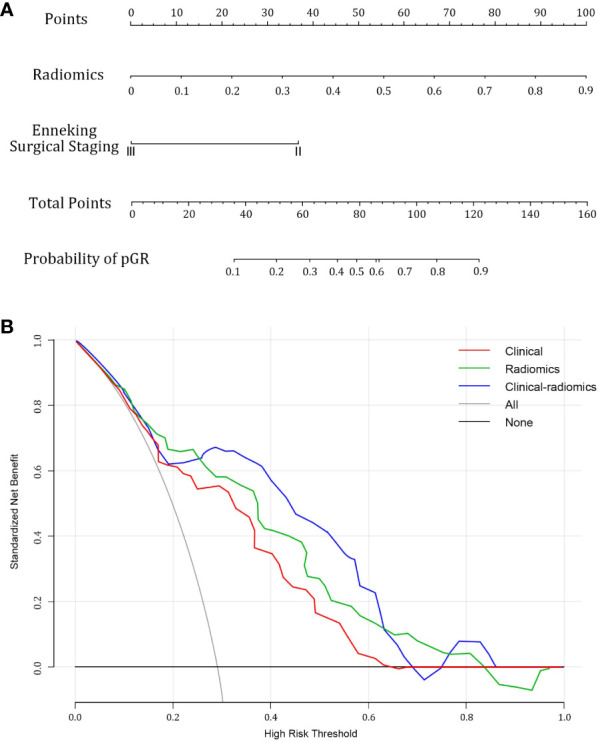
Combined clinical-radiomics nomogram and its utility. **(A)** A nomogram integrating the radiomics score and clinical variables was constructed **(B)** Decision curve analysis curves for clinical, radiomics, and combined nomogram models in the whole dataset showed that the nomogram had favorable clinical utility.

Following univariate and multivariate analyses sequentially, surgical staging was selected out to construct the nomogram. The nomogram model was constructed using a multivariate logistic regression that included surgical staging and the radiomics signature, which was then visually represented in a nomogram (**Figure 5A**). Of the two factors, the radiomics signature held the most weight in predicting outcomes, as shown by its extended scale, followed by surgical staging. The nomogram’s scoring system correlated the high and low probability segments of the radiomics label with corresponding scores on the axis. Summing each factor’s scores provided a total score, which translated into an individual’s probability of effective chemotherapy on the probability axis. The nomograms had AUCs of 0.887 (95% CI, 0.759–0.944) in the training set and 0.848 (95% CI, 0.669–0.931) in the test set, respectively. The ROC curves showed the predictive probabilities of the nomogram outperformed the clinical model, radiomics model, and traditional imaging parameters (ADC values) (**Figures 3A–D**). The Decision Curve Analysis (DCA) for the nomogram across the entire dataset demonstrated strong clinical utility (**Figure 5B**). In the test set, the nomogram provided more benefit than treating all or no patients when the threshold probability of a good response (pGR) ranged from 0.11 to 0.61 and 0.65 to 0.84. The nomogram also outperformed the radiomics model and clinical model within these threshold ranges. The DeLong test indicated significant differences between the clinical-radiomics nomogram model and the clinical model, as well as between the radiomics model and the clinical model in the training set (p < 0.05) (**Table 3**).

**Table 3:** Results of DeLong test between clinical model, radiomics model and nomogram model.

	Clinical vs Radiomcs *P value*	Clinical vs Nomogram *P value*	Radiomcs vs Nomogram *P value*
Training set	0.036	0.004	0.085
Test set	0.474	0.231	0.347

## 4 Discussion

In this study, we developed a combined clinical-radiomics nomogram based on MRI-DWI to preoperatively predict the response to NAC in osteosarcoma patients. The nomogram incorporated features extracted from DWI images of segmented lesions alongside objective clinical features. It outperformed the traditional MRI ADC value and standalone radiomics model, as evidenced by decision curve analysis (DCA) illustrating its clinical utility.

Osteosarcoma is notorious for its aggressiveness and poor prognosis. Despite the significant improvements in 5-year survival rates achieved by integrating NAC with surgical resection, about 30-40% of patients still suffer from local recurrence or distant metastasis, which drastically reduces the 5-year survival rate to 23-29% (14). Traditional imaging assessments like CT and MRI evaluate chemotherapy effects based on morphological changes, including tumor volume, internal liquefactive necrosis, calcification, and surrounding bone destruction. However, these methods fall short in reflecting the viability of tumor cells and correlating with tumor tissue necrosis rates, thus limiting their effectiveness in grading, evaluating treatment response, and predicting the prognosis of osteosarcoma (15). Additionally, research suggested that changes in tumor volume are not significantly linked to chemotherapy response. Imaging techniques such as dynamic contrast-enhanced, PET/CT, and DWI (16) offered valuable insights into tumor angiogenesis and cellular activity, showing great promise (17–19). Nevertheless, the heterogeneity of tumors presents a challenge in identifying highly specific quantitative markers, creating an urgent clinical need for a straightforward, accurate, and reproducible method to evaluate the effectiveness of NAC. Such a tool would facilitate early identification of tumor chemosensitivity, allow timely adjustments to chemotherapy and surgical plans, and predict preoperative chemotherapy efficacy and prognosis.

Kumar and colleagues (20) advanced the field of radiomics by extracting a broad array of high-dimensional quantitative imaging features from routine medical images, such as MRI, PET, and CT, in a high-throughput manner. This approach quantitatively characterizes the temporal and spatial heterogeneity within the images, uncovering features that are beyond human visual perception. Radiomics research typically involves five steps: image acquisition, ROI segmentation, radiomic feature extraction, feature selection and dimensionality reduction, and predictive model performance evaluation (21). By tapping into the rich digital information from conventional medical images and utilizing machine learning algorithms, radiomics reduces observer subjectivity and offers a quantitative analysis of tumor heterogeneity, which is invaluable for precise disease diagnosis and treatment. There has been a study constructing MRI T2WI-based radiomics models to predict chemotherapy response in osteosarcoma, such as the work by Jingyu Zhong et al. (22). The ultimate aim of radiomics is to devise models with strong predictive accuracy, applicable to both existing and new data sets. The clinical value of radiomics is increasingly recognized across various studies as an emerging technology.

Our study introduced a radiomics model based on MRI-DWI images that did not require a contrast agent, offered a wealth of biologically relevant information, including tumor cellular density, and was conducted within a shorter scanning period. Unlike prior studies that focused exclusively on radiomics parameters for model construction, our research integrated objective clinical features with radiomic scores, thereby enhanced the model’s predictive accuracy and clinical applicability. The final radiomics model selected 8 textural features strongly related to NAC response (23, 24). Six of these features were from the Gray Level Dependence Matrix (GLDM), and two from the Gray Level Size Zone Matrix (GLSZM), suggesting that patterns in gray values hold significance in predicting NAC effectiveness. This notion was consistent with previous imaging studies that explored the prediction of sentinel lymph node metastasis in breast cancer, highlighting the potential of these gray value patterns in clinical prognosis.

In this study, we employed the potential of radiomics to preliminarily evaluate the effectiveness of a nomogram model that integrated MRI DWI radiomics and clinical features for predicting the response to NAC in osteosarcoma. After conducting a rigorous statistical analysis and reducing the dimensionality of all extracted radiomic features, we identified eight features with significant predictive value. These were then utilized to construct a combined nomogram model. The model showcased promising predictive performance in the test set, with an AUC of 0.848 (95% CI: 0.668-0.951), a sensitivity of 75%, and a specificity of 73%. These results suggested that the combined nomogram model we derived had considerable potential in predicting the outcome of NAC in osteosarcoma.

The combined nomogram model, with its high predictive accuracy, presented as both user-friendly and practical, offering a broad spectrum of clinical applications in managing osteosarcoma. This model was instrumental in refining treatment strategies, particularly for locally advanced cervical cancer. Presently, the lack of effective biomarkers to predict chemotherapy efficacy renders the decision to administer preoperative NAC somewhat arbitrary. The utilization of this nomogram empowers physicians to discern which patients are likely to be responsive or resistant to chemotherapy prior to commencing treatment, thereby guiding the selection of suitable therapeutic regimens. For individuals with extensive tumor burden who exhibit resistance to chemotherapy, prompt consideration of concurrent chemoradiotherapy may be appropriate. Conversely, for younger patients who are responsive to chemotherapy, pursuing NAC followed by surgical intervention is a feasible approach. Moreover, in regions where access to radiotherapy is limited, particularly some developing countries, NAC is being considered as a precursor to concurrent chemoradiotherapy for tumor management. The combined radiomics model also holds promise for identifying optimal candidates for NAC in preparation for concurrent chemoradiotherapy. Additionally, the nomogram could potentially forecast the response of patients with recurrent or metastatic cervical cancer to systemic chemotherapy, based on preoperative MRI images. Should this combined radiomics nomogram be integrated into clinical practice, it stands to make a substantial contribution to the evolution of personalized precision medicine for osteosarcoma. To validate its clinical applicability, we conducted decision curve analysis (DCA), a methodology corroborated by findings from four preceding studies (25–27).

## 5 Shortcomings of this study

① This study utilized only one MRI scanning sequence; combining different sequences is expected to enhance predictive performance. ② The ROI delineation for this study was performed manually, a process that is both laborious and time-intensive, rendering it impractical for handling large datasets and subject to variability due to the irregular contours of tumor lesions. This variability could influence the precision of feature selection as well as the repeatability and reliability of the model. Hence, exploration into automated ROI segmentation methods is justified; ③ The study’s focus was restricted to assessing the efficacy of radiomics features from a single pre-treatment time point in predicting the NAC response in osteosarcoma, without considering sequential imaging during the treatment course or carrying out an exhaustive comparison with other MRI scanning modalities. Addressing these considerations will be central to future research and validation efforts.

## 6 Conclusion

To conclude, the findings of our study indicated that the integrated model combining radiomics and clinical features demonstrates robust predictive efficacy for predicting the response to neoadjuvant chemotherapy in osteosarcoma. This model is poised to offer invaluable insights for clinical decision-making in the diagnosis and treatment of osteosarcoma, thus advancing the pursuit of personalized and precision medicine for this challenging disease.

## Data availability statement

The original contributions presented in the study are included in the article/supplementary material. Further inquiries can be directed to the corresponding author.

## Ethics statement

The studies involving humans were approved by Ethics Committee of Henan Provincial People’s Hospital. The studies were conducted in accordance with the local legislation and institutional requirements. Written informed consent for participation in this study was provided by the participants’ legal guardians/next of kin.

## Author contributions

LZ: Writing – original draft, Writing – review & editing, Data curation, Funding acquisition, Investigation, Project administration, Visualization. QG: Data curation, Investigation, Methodology, Writing – original draft. YD: Resources, Supervision, Validation, Writing – review & editing. TC: Data curation, Investigation, Methodology, Supervision, Validation, Writing – review & editing. YX: Data curation, Investigation, Methodology, Software, Writing – review & editing. HL: Writing - review & editing, Data curation. SG: Funding acquisition, Supervision, Validation, Writing – review & editing.
